# Supplementary material for: Optimal Design of THEDES Based on Perillyl Alcohol and Ibuprofen
Source: Pharmaceutics. 2020 Nov 20;12(11):1121. doi: 10.3390/pharmaceutics12111121 (PMC7699764; doi:10.3390/pharmaceutics12111121)
Supplement: Supplementary file 1 [file pharmaceutics-12-01121-s001.pdf]

pharmaceutics

MDPI

Supplementary Materials: Therapeutic Roles of Deep Eutectic Systems based on Perillyl Alcohol and Ibuprofen

Eduardo Silva, Filipe Oliveira, Joana M. Silva, Ana Matias, Rui L. Reis, Ana Rita C. Duarte

Table S1. Representative images of disk diffusion assay plates for individual compounds and eutectic blends regarding *Staphylococcus aureus* (Gram-positive) ATCC 25923.

**Isolated compounds**	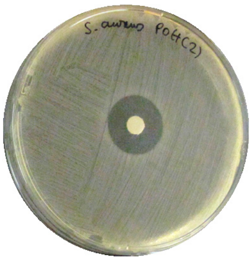	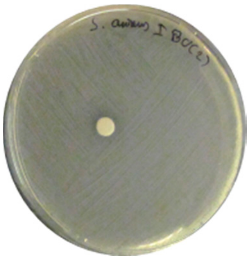	
	**Perillyl alcohol (POH)**	**Ibuprofen (IBU)**	
**THEDES**	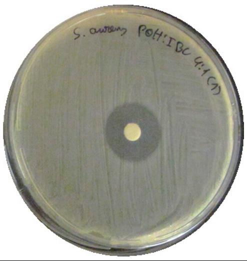	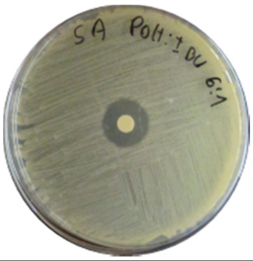	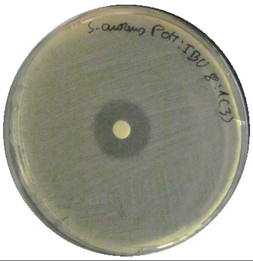
	**POH:IBU 4:1**	**POH:IBU 6:1**	**POH:IBU 8:1**
**Controls**	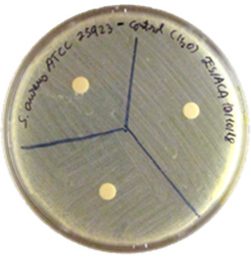	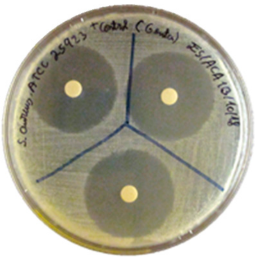	
	**Sterile water (– control)**	**Gentamicin (+ control)**	

Table S2. Representative images of disk diffusion assay plates for individual compounds and eutectic blends regarding *Staphylococcus aureus* (Gram-positive) ATCC 700698 (MRSA).

**Isolated compounds**	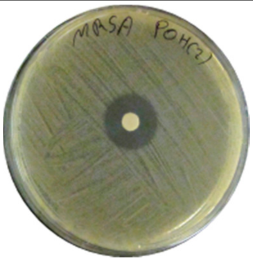	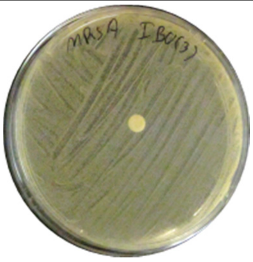	
	**Perillyl alcohol (POH)**	**Ibuprofen (IBU)**	
**THEDES**	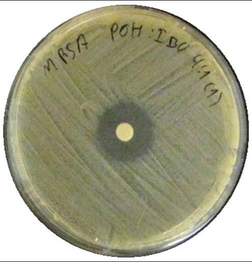	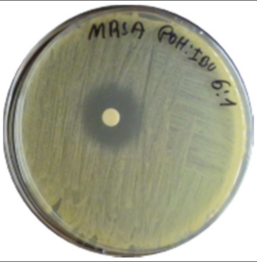	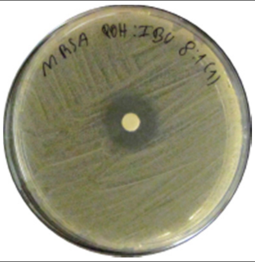
	**POH:IBU 4:1**	**POH:IBU 6:1**	**POH:IBU 8:1**
**Controls**	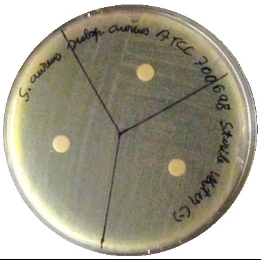	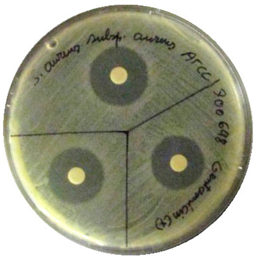	
	**Sterile water (– control)**	**Gentamicin (+ control)**	

Table S3. Representative images of disk diffusion assay plates for individual compounds and eutectic blends regarding *Staphylococcus epidermis* (Gram-positive) ATCC 35984 (MRSE).

**Isolated compounds**	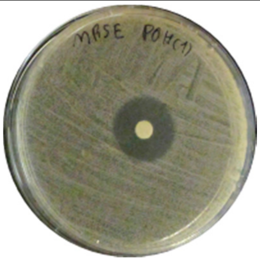	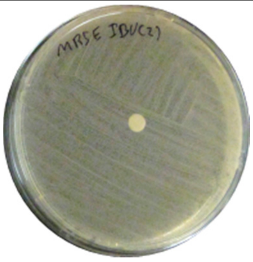	
	**Perillylalcohol (POH)**	**Ibuprofen (IBU)**	
**THEDES**	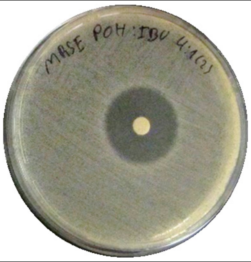	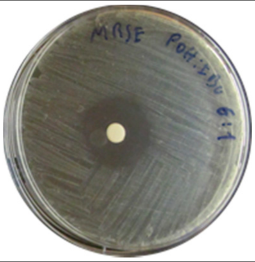	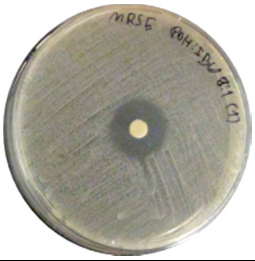
	**POH:IBU 4:1**	**POH:IBU 6:1**	**POH:IBU 8:1**
**Controls**	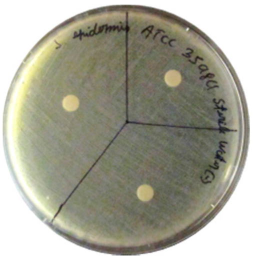	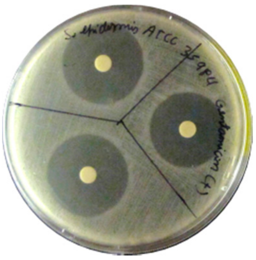	
	**Sterile water (– control)**	**Gentamicin (+ control)**	

Table S4. Representative images of disk diffusion assay plates for individual compounds and eutectic blends regarding *Pseudomonas aeruginosa* (Gram-negative) ATCC 27853.

<table>
<tr><td rowspan="2">Isolated compounds</td><td colspan="3">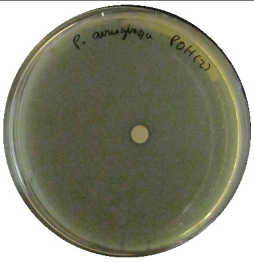</td><td colspan="3">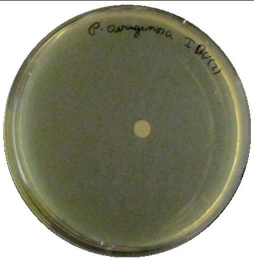</td></tr>
<tr><td colspan="3">Perillyl alcohol (POH)</td><td colspan="3">Ibuprofen (IBU)</td></tr>
<tr><td rowspan="2">THEDES</td><td colspan="2">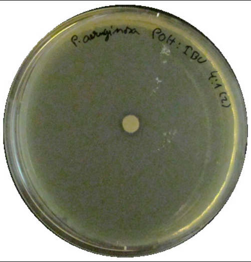</td><td colspan="2">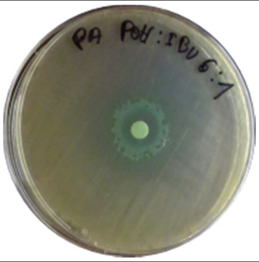</td><td colspan="2">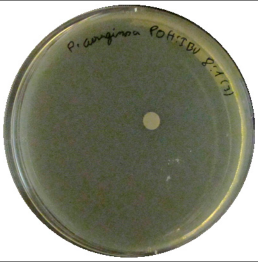</td></tr>
<tr><td colspan="2">POH:IBU 4:1</td><td colspan="2">POH:IBU 6:1</td><td colspan="2">POH:IBU 8:1</td></tr>
<tr><td rowspan="2">Controls</td><td colspan="3">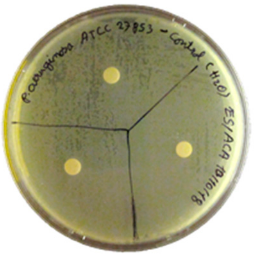</td><td colspan="3">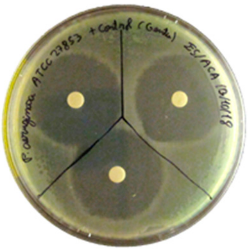</td></tr>
<tr><td colspan="3">Sterile water (– control)</td><td colspan="3">Gentamicin (+ control)</td></tr>
</table>

Table S5. Representative images of disk diffusion assay plates for individual compounds and eutectic blends regarding *Escherichia coli* (Gram-negative) ATCC 25922.

**Isolated compounds**	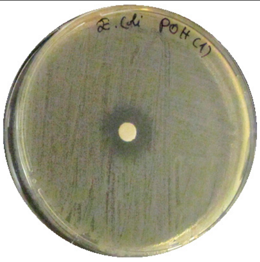	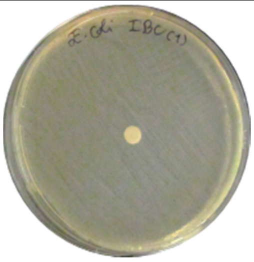	
	**Perillyl alcohol (POH)**	**Ibuprofen (IBU)**	
**THEDES**	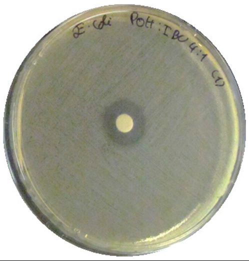	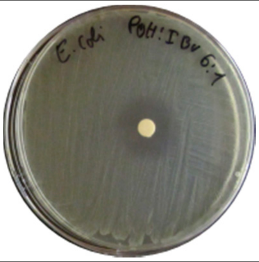	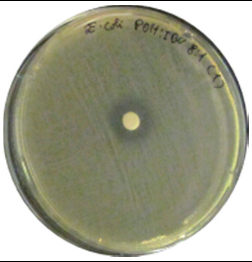
	**POH:IBU 4:1**	**POH:IBU 6:1**	**POH:IBU 8:1**
**Controls**	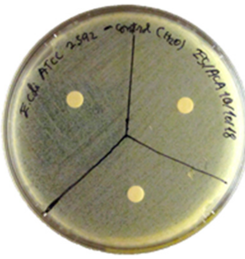	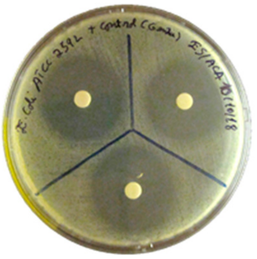	
	**Sterile water (− control)**	**Gentamicin (+ control)**	

Table S6. Representative images of disk diffusion assay plates for individual compounds and eutectic blends regarding *Candida albicans* (yeast) ATCC 90029.

**Isolated compounds**	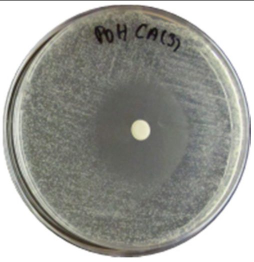	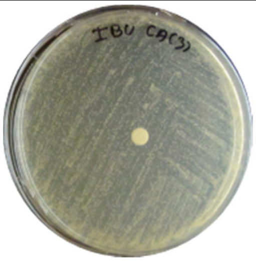	
	**Perillyl alcohol (POH)**	**Ibuprofen (IBU)**	
**THEDES**	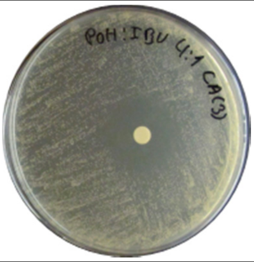	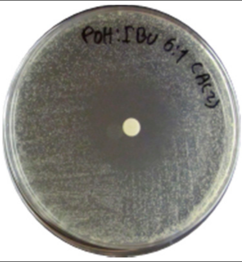	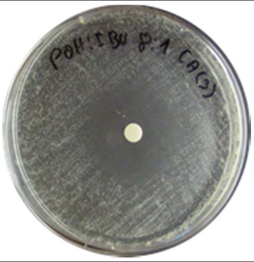
	**POH:IBU 4:1**	**POH:IBU 6:1**	**POH:IBU 8:1**
**Controls**	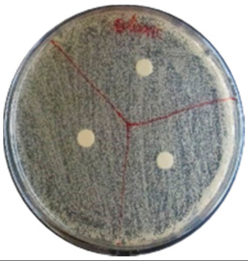	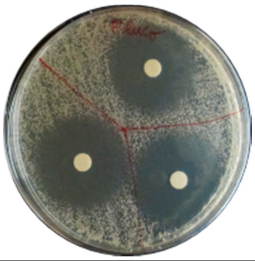	
	**Sterile water (– control)**	**Gentamicin (+ control)**	

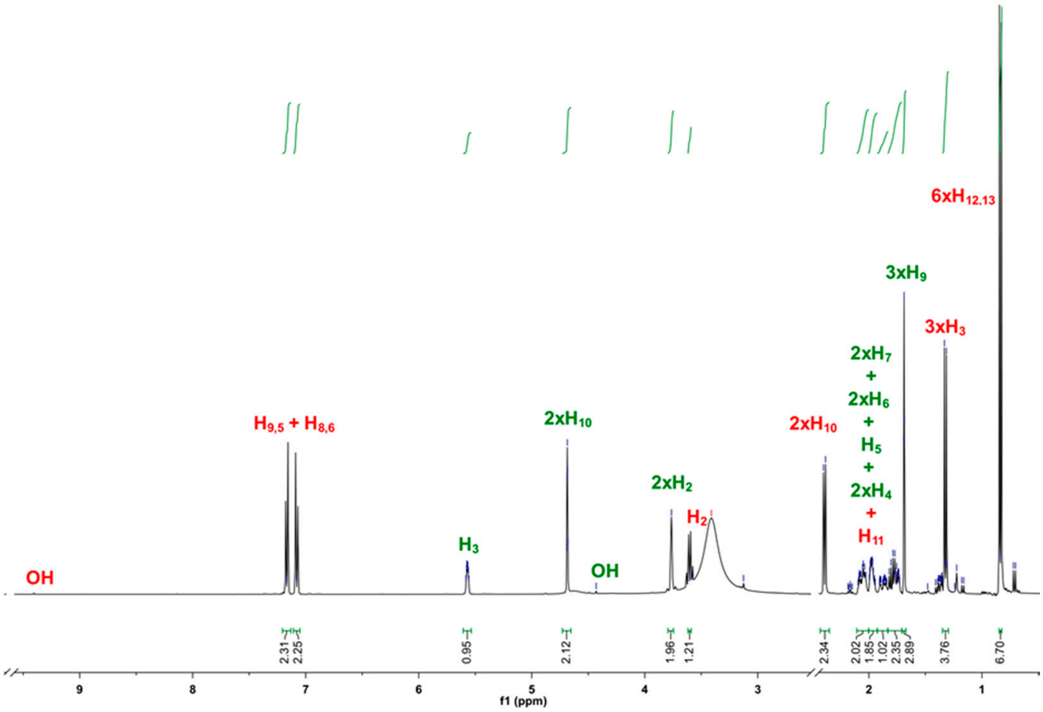

Figure S1. ^1H NMR spectra of POH:IBU 1:1. Peak assignment and integration were fully performed.

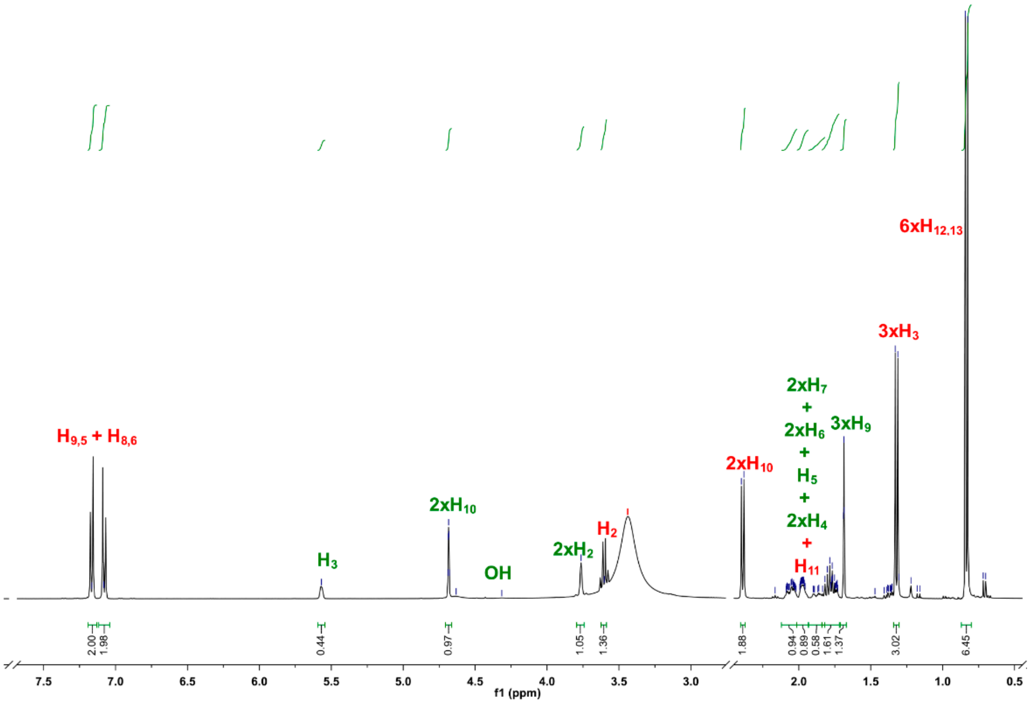

Figure S2. ^{1}H NMR spectra of POH:IBU 1:2. Peak assignment and integration were fully performed.

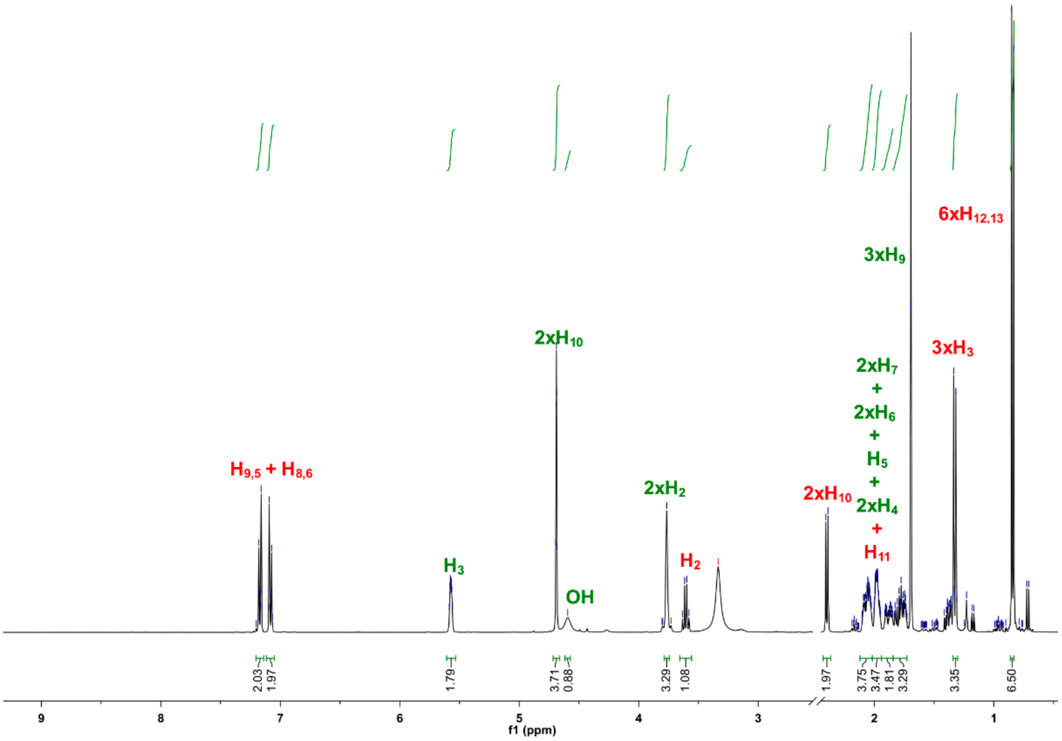

Figure S3. ^{1}H NMR spectra of POH:IBU 2:1. Peak assignment and integration were fully performed.

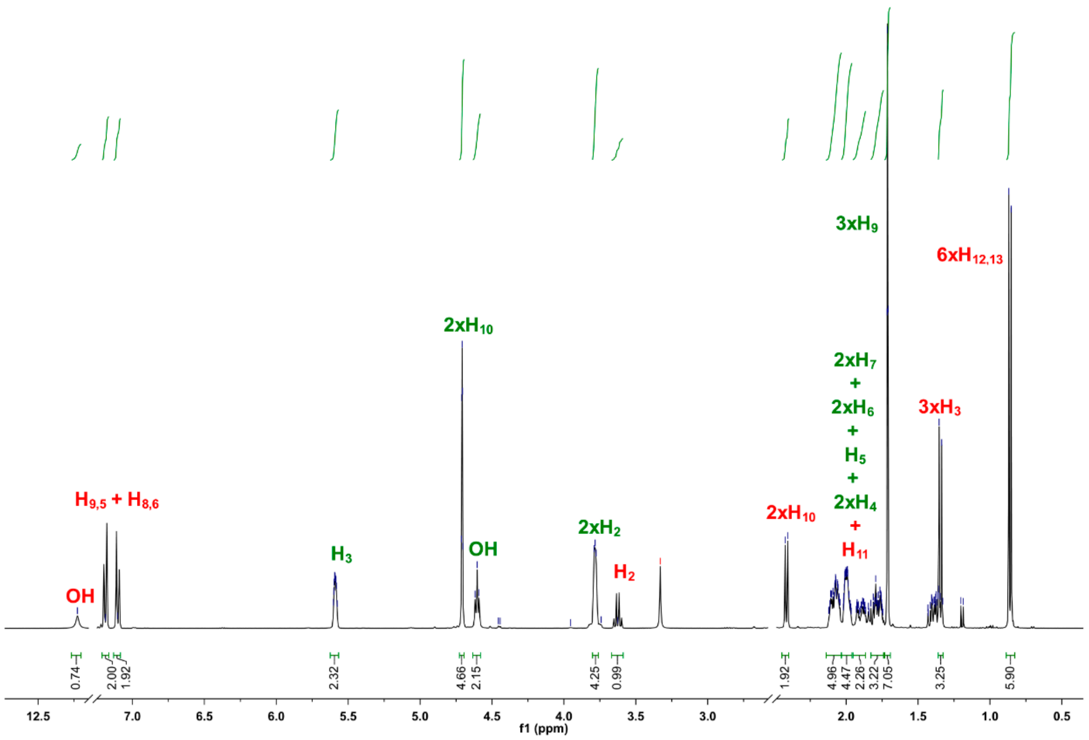

Figure S4. ^{1}H NMR spectra of POH:IBU 3:1. Peak assignment and integration were fully performed.

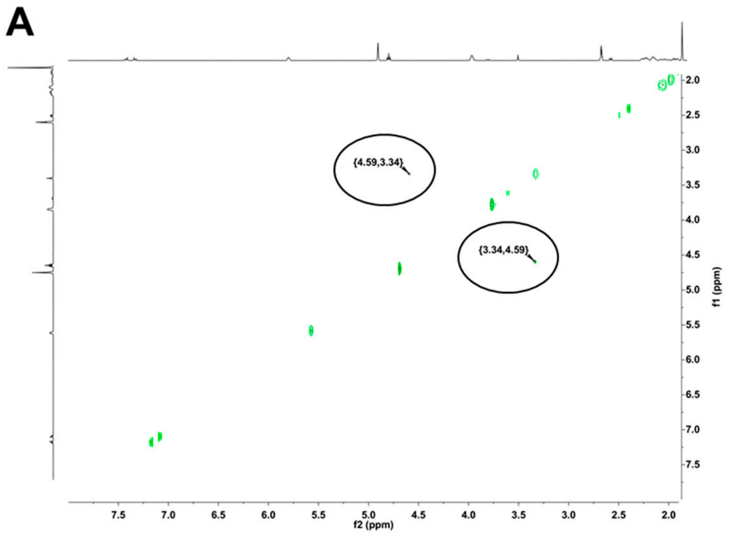

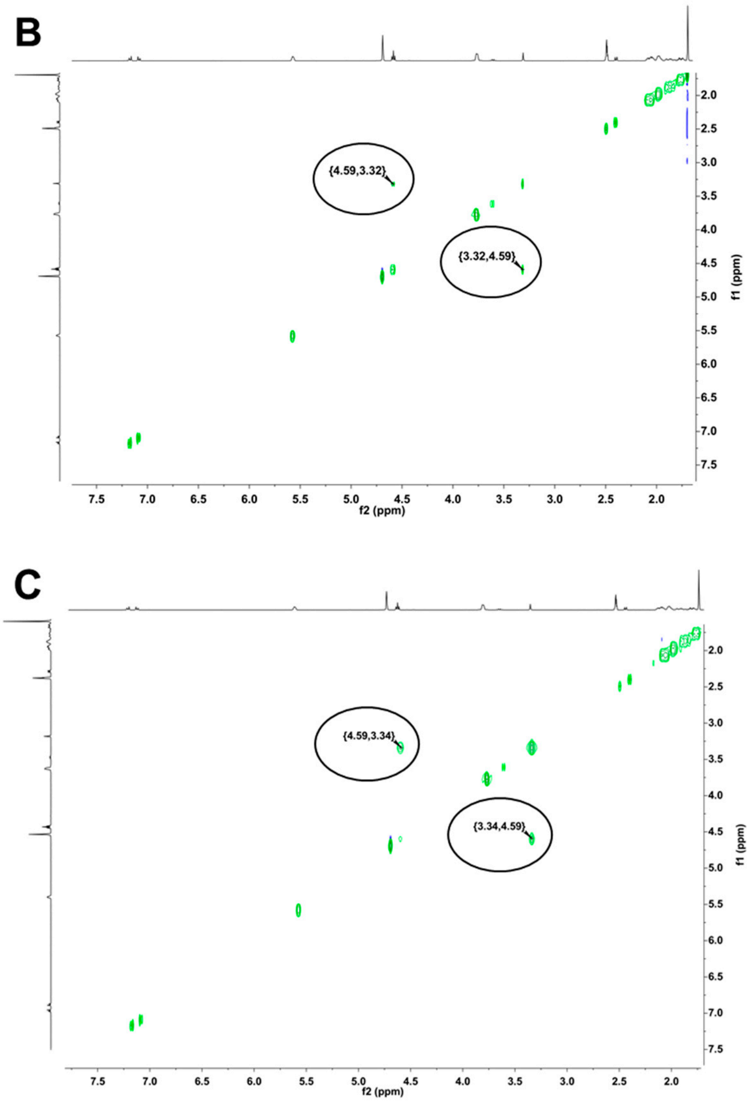

Figure S5. NOESY spectra of THEDES (**A**) POH:IBU 4:1, (**B**) POH:IBU 6:1 and (**C**) POH:IBU 8:1.
